# Paediatric Renal Tumors: A State-of-the-Art Review

**DOI:** 10.1007/s11912-025-01644-8

**Published:** 2025-02-07

**Authors:** Cecilia Salzillo, Gerardo Cazzato, Gabriella Serio, Andrea Marzullo

**Affiliations:** 1 Department of Precision and Regenerative Medicine and Ionian Area, Pathology Unit, University of Bari “Aldo Moro”, Piazza Giulio Cesare 11, 70121 Bari, Italy; 2 Department of Experimental Medicine, PhD Course in Public Health, University of Campania “Luigi Vanvitelli”, Luciano Armanni 5, 80138 Naples, Italy

**Keywords:** Paediatric renal tumors, Pediatric kidney tumors, Pathology, Systemic therapy, Immunotherapy, Targeted therapy

## Abstract

**Purpose of Review:**

Pediatric renal tumors comprise a wide range of conditions, both malignant and benign, that affect children and require a multidisciplinary approach for optimal diagnosis and treatment. This review offers an in-depth analysis of the epidemiology, diagnosis, treatment options, outcomes, and survival of major pediatric renal tumors.

**Recent Findings:**

Wilms tumor, or nephroblastoma, is the most common form of renal tumor in children, characterized by growth from primitive renal cells. Standard treatment involves a combination of surgery, chemotherapy and, in some cases, radiation therapy, with the aim of removing the tumor, preventing recurrence and maximizing the chances of long-term recovery. Less common pediatric renal tumors, such as renal clear cell sarcoma, renal cell carcinoma, mesoblastic nephroma, and malignant rhabdoid tumor, require similarly careful and individualized management. Therapeutic strategies, which depend on the characteristics of the tumor, the stage of the disease and the individual response to therapy, may include surgery, chemotherapy, radiotherapy and, in some cases, molecular targeted therapies, immunotherapies and genetic and epigenetic therapies.

**Summary:**

The management of pediatric kidney tumors requires the involvement of a multidisciplinary team of specialists to ensure accurate evaluation, optimal treatments and long-term follow-up. The aim is to maximize the prospects for recovery and improve the quality of life of patients and their families. Advances in innovative, personalized therapies represent an important opportunity to further improve clinical outcomes in these patients.

## Introduction

The pediatric renal tumors (PRTs) include benign and malignant tumors that affect the kidney in childhood, constituting 3.2-11.1% of pediatric tumors worldwide [1–3], and according to other studies 7% of pediatric tumors [4] and ~ 6% of all pediatric cancers [5].

PRTs include Wilms tumor (WT) and non-Wilms tumor (N-WT) such as renal cell carcinoma (RCC), clear cell sarcoma of the kidney (CCSK), mesoblastic nephroma (MN), malignant rhabdoid tumor (MRT), and other rarer tumors such as soft tissue sarcomas/PNET, neuroblastoma of the kidney, non-Hodgkin lymphoma (primary site in the kidney), teratoma and angiomyolipoma.

The vast majority of PRTs approximately between 80% and 90% [4, 5] are represented by WT, and approximately 30% of affected patients have survival rates below 70% [4].

Specifically, WT survival has improved greatly in recent decades; 5-year estimates in the general population have risen from approximately 75% around 1980 to 85–90% at the turn of the century [6]. Since the increase, survival in Europe has stabilized at 90% [7].

In contrast, other malignant renal tumors defined as non-WT tend to have less favorable outcomes. A population-based European study reported 5-year survival estimates of 23% for MRTK, 68% for CCSK, and 87% for RCC in children diagnosed in the 1990s [7].

In fact, high-risk renal tumors, which tend to have less favorable outcomes, include MRT, anaplastic WT, stage IV rhabdoid tumor of the kidney, several subtypes of RCC such as clear cell, papillary, chromophobe and ductal Bellins [4].

It is therefore clear that the most important prognostic marker for PRTs is tumor histology, followed by tumor stage, which are fundamental to direct treatment with a multidisciplinary approach.

To standardize the diagnosis and therefore the treatment, based on the results of previous national and international studies and trials, the Renal Tumor Study Group of the International Society of Pediatric Oncology (SIOP-RTSG) has developed a new study protocol for pediatric renal tumors called the UMBRELLA protocol SIOP-RTSG 2016 or briefly the UMBRELLA protocol [5].

This protocol allows the treatment to be adapted to patients with both poor and excellent prognoses. The strategy requires patient-centered assessment and increased parent and patient involvement.

The UMBRELLA protocol [5], based on the 2013 UK IMPORT protocol and the experiences of the 2001 SIOP, aims to identify and refine new biomarkers for the management of children, adolescents and young adults with Wilms tumor and other non-Wilms pediatric renal tumors (Table 1).

**Table 1:** Pediatric renal tumors

Type of Tumor	Characteristics	Prognosis	Treatment
*Wilms tumor*			
Wilms tumor	Most common renal cancer in children	Variable, usually good with early treatment	Depending on the stage and histological features: surgery, chemotherapy, radiotherapy and new therapeutic
Anaplastic Wilms tumor	More aggressive variant of Wilms tumor with anaplastic cells	Unfavorable	Surgery, intensive chemotherapy, radiotherapy
*Non-Wilms Tumor*			
Renal clear cell sarcoma	Second most frequent renal malignant neoplasm	Unfavorable, it is more aggressive than Wilms tumor	Depending on the stage: surgery, chemotherapy and radiotherapy
Renal cell carcinoma	Very rare in children	It varies depending on the histotype and stage	Depending on the stage and histological features: surgery, chemotherapy, radiotherapy and new therapeutic
Malignant rhabdoid tumour of the kidney	Rare and aggressive	Very unfavorable	Surgery, chemotherapy, radiotherapy
Congenital Mesoblastic Nephroma	Most frequent renal tumor in the fetus and newborn	Variable, usually good	Surgery and chemotherapy

## Materials and Methods

Our state-of-the-art review is a review of the pathology and treatment of Paediatric Renal Tumors, addressing the topic from pathogenesis to therapy and focusing on the importance of differential diagnosis for the best therapeutic approach.

The review was conducted in PubMed, Scopus and WOS with the following search keywords “paediatric renal tumor” OR “pediatric kidney tumors” OR “Wilms tumor” OR “Non-Wilms tumor” OR “renal clear cell sarcoma” OR “renal cell carcinoma” OR “malignant rhabdoid tumour of the kidney” OR “congenital mesoblastic nephroma”, with inclusion criteria primary studies, secondary studies and English language. Additionally, Google Schoolar was used for gray literature.

## Wilms Tumor

Wilms tumour (WT), or nephroblastoma, is the most frequent renal tumour in children and was first described in 1899 by the German doctor Max Wilms [8].

WT is an embryonal malignancy resulting from disrupted kidney development [9]. During embryogenesis, the intermediate mesoderm forms the metanephric mesenchyme, which transforms into epithelium to create the kidney cells [10]. In the WT, this process is disrupted at various levels, generating mixtures of blastemal, epithelial, and stromal cells, sometimes with myogenic differentiation [11]. The histology of the tumour reflects both genetic defects and times of divergence from normal renal formation [10].

The cause of WT is not completely clear but is thought to be linked to genetic changes in the normal development of the genitourinary tract [8].

Initially, only mutations of WT1, CTNNB1, WTX and loss of H19-IGF2 imprinting were known, explaining only some cases. Other genes associated with WT include TP53 and MYNC [12]. Furthermore, loss of heterozygosity in chromosomes 1p, 1q, 11p15 and 16q may indicate aggressive clinical behaviour [13] but does not clarify the mechanisms of tumor development nor the possible therapeutic targets. Recent analyses have uncovered additional genetic factors, including chromatin modifiers and microRNA genes such as DROSHA, DGCR8, DICER1 and other, involved in renal development [14]. The mutation of these genes is important for therapeutic implications.

WT is the most frequent type of renal tumor in children, but its incidence varies greatly between different regions and ethnic groups [10].

Specifically, in East Asia the annual incidence is lower with 4.3 cases per million children, compared to North America and Europe where 8–9 cases per million are registered [15]. In the United States, African American children have the highest incidence with 9.7 cases per million, while those in the Asia-Pacific Islands have the lowest incidence with 3.7 cases per million [15]. However, estimating the global incidence is difficult due to the lack of childhood cancer registries in some regions and the poor quality of available data, as not all cancers are reported or registered [16]. Furthermore, in areas with fewer resources, around 50% of patients are diagnosed when the cancer has already metastasized [17].

WT is one of the rare childhood cancers that is most frequently found in girls, with a prevalence approximately 10% higher than in boys. In boys, the incidence of this cancer reaches its peak at 1 year of age, with 17.9 cases per million person-years. In girls, however, the incidence remains nearly constant between 1 and 3 years of age, with rates of 17.8, 18.0 and 18.1 cases per million person-years respectively [10]. 

WT usually manifests as a single lesion, but in approximately 7% of cases it may be multifocal, and in 5–9% of cases it is bilateral [15]. Generally, tumors affecting only one kidney develop at a slightly older age than those affecting both kidneys.

17% of WTs present as part of a well-defined malformation syndrome, and among these, approximately 10% are linked to a specific genetic predisposition for this type of tumor [10].

The genetic syndromes to which WT is associated are WAGR syndrome with a 50% chance of developing the tumor, Denys-Drash syndrome with a 90% chance, Beckwith-Wiedemann syndrome with a 5–10 chance %, and other less common syndromes such as Sotos syndrome, Perlman syndrome, trisomy 18, Frasier syndrome, Bloom syndrome, Li-Fraumeni syndrome and Simpson-Golabi-Behmel syndrome [8].

Clinically, in most children, WT manifests as a palpable asymptomatic abdominal mass that is first noticed by chance by a family member or primary care physician. Other symptoms present in 20–25% are hypertension, hematuria, and flank pain, and presentation after blunting abdominal trauma with abdominal/side pain and blood loss has also been observed [18].

Subsequently, to confirm the diagnosis of WT it is essential to proceed with imaging tests and laboratory tests.

An abdominal ultrasound (US) is often the first test used to identify a kidney mass. If necessary, a magnetic resonance imaging (MRI) or computed tomography (CT) scan may also be done to get more precise images and to assess whether the tumor has spread to the lymph nodes or other organs. A chest X-ray (XR) may be done to check for metastases in the lungs, as this is the first site of metastases.

Laboratory tests evaluate renal function, electrolyte levels and complete blood count, urinalysis, coagulation studies, and cytogenetic studies for the search for 1p and 16q deletion.

In some cases, a renal biopsy is required to confirm the diagnosis of WT, to administer appropriate pre-operative chemotherapy, and some tissue is stored frozen for molecular studies. However, the diagnosis is often made on the basis of clinical and radiological characteristics, and the tumor is surgically removed.

Staging of the tumor is critical to understanding how far the cancer has spread and to planning treatment. The National Wilms Tumor Study Group (NWTSG) staging system is commonly used, classifying the tumor from Stage I, which is limited to the kidney and completely removed, to Stage V, which is present in both kidneys.

### Pathology

#### Sampling

The nephrectomy specimen is photographed, measured, and inked. After opening, samples of tumor and normal kidney tissue are taken for biological studies. At least one longitudinal slice of the cancer is sampled, with additional blocks from different areas. In the case of a multicentric tumor, each nodule is sampled. The interface between tumor and normal kidney, renal and tumor capsule, and renal sinus involvement are examined for staging. The remaining kidney, hilar fat, and all lymph nodes are also sampled for metastases.

#### Macroscopy

Macroscopically, WT usually presents as large masses altering renal contours, varying significantly in size.

**Fig. 1:**
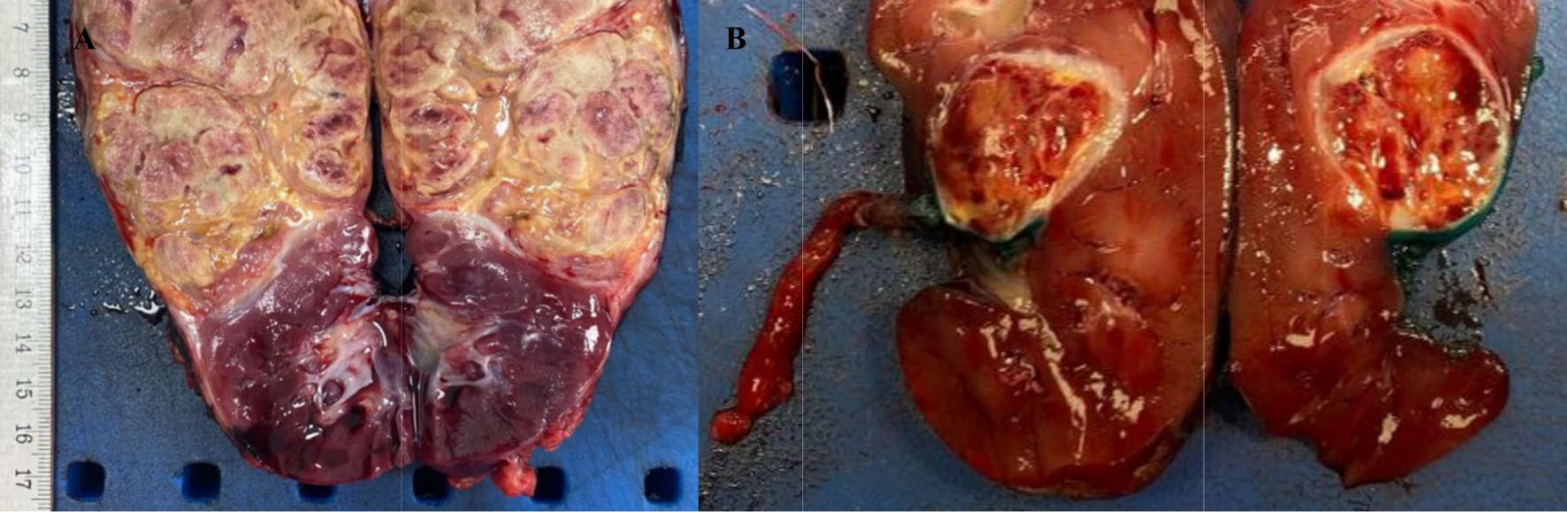
Wilms tumor (**A and B**) On section, the surface is heterogeneous, with areas of viable tumor, hemorrhage, and necrosis

Upon cutting, the surface is heterogeneous in many cases, with areas of viable tumor, hemorrhage, and necrosis (Fig. 1), especially in pretreated specimens. The viable tumor is usually light gray to slightly pink solid in consistency or yellow grey in soft consistency. Some tumors are cystic and careful investigation for solid foci is necessary.

To avoid contamination, it is important to sample the hilar margins, including vessels, if possible before cutting the tumor. Multicentric tumors are present in 5% and are usually associated with nephrogenic rests [19, 20].

#### Histology

WT classically presents a triphasic histological pattern, composed of epithelial, stromal and blastemal components, and the proportions and degree of differentiation can vary, giving rise to a large variety of tumor presentations. Biphasic and monophasic variants are less frequent.

Preoperative chemotherapy, according to the protocol of the International Society of Pediatric Oncology (SIOP), can considerably modify the original histology of the tumor, reducing or increasing some elements or inducing their maturation.

The blastema is the least differentiated and most malignant component of WT, characterized by small, round blue cells with overlapping nuclei and high mitotic activity. There are different histological models of blastema: diffuse, serpentine, nodular and basaloid. The serpentine pattern presents broad bands of undifferentiated cells surrounded by fibromyxoid stroma, whereas in the basaloid variant the blastema cords have a peripheral palisade of elongated cells with epithelial differentiation.

All these patterns can coexist in the same tumor and have no prognostic value, but their recognition is useful to differentiate the tumor from other “small round blue cell tumors” when only the blastemal component is present.

Blastemal-type WT, often with diffuse growth, may present marked infiltration without a pseudocapsule.

Primitive tubular epithelial structures in blastemal nodules may appear to resemble neuroblastoma with pseudorosettes. Additionally, the appearance of epithelioid or spindle cells may vary depending on early differentiation. There are no definitive criteria to distinguish blastema from early epithelial differentiation or the stromal lineage, and classification of WT subtypes is based on subjective morphological criteria.

The epithelial component of WT displays various stages of differentiation, ranging from primitive epithelial rosette-like structures to tubules and glomeruli-like structures, reflecting different stages of nephrogenesis. Examples of heterologous differentiation include squamous epithelial islands and mucinous epithelia.

The stromal component may include both densely packed undifferentiated mesenchymal cells and looser myxoid cell areas, which may or may not be difficult to distinguish from chemotherapy-modified nontumorous stroma. In some tumors, especially after preoperative chemotherapy, a heterologous differentiation of the neoplastic stroma, which includes smooth or skeletal muscle cells, adipose tissue, cartilage, bone and even glial tissue, can be observed.

Chemotherapy-induced modification may include necrosis, hemorrhage, fibrosis, and hemosiderin-laden macrophages. The blastemal component, highly proliferative, responds well to chemotherapy, while the mature epithelial and stromal components are less sensitive and show a reduced response to preoperative therapy.

It is important to highlight that classification and assessment of response to chemotherapy vary between SIOP and NWTS/COG, influencing risk stratification and treatment. Fully necrotic tumors are considered low risk, while tumors with more than 10% blastema are classified as mixed [19, 21].

Furthermore, the SIOP uses the terms “stromal type” or “epithelial type” to classify pretreated tumors, whereas the NWTS/COG describes them as “stromal-predominant” or “epithelial-predominant.”

Anaplastic WT (AWT) constitutes 5–7% of cases of this type of renal tumor [22]. The diagnosis of anaplasia is based on the presence of large, atypical mitotic figures, and enlarged, hyperchromatic nuclei.

These tumors are typically aneuploid, and the anaplasia may be focused or diffuse. Focal anaplasia indicates a limited area with anaplastic characteristics, while diffuse anaplasia is considered an unfavourable histological feature and can negatively influence the prognosis [22].

Subsequently, SIOP introduced a new risk stratification, and focal AWT was classified as intermediate-risk WT, whereas diffuse AWT remained a high-risk tumor [23].

AWT often express the protein p53 and have mutations in the TP53 gene, which are associated with a worse prognosis. MYCN gene dysregulation has also been associated with unfavourable outcomes in cases of anaplastic histology [19].

Nephrogenic rests (NRs) are abnormal areas of embryonic tissue that persist beyond 36 weeks of development and are present in 30–44% of WT kidneys [19]. The term “nephroblastomatosis” was introduced in 1961 and indicates a lesion composed of immature renal tissue.

There are two major types of NR: perilobar (PLNR) and intralobar (ILNR). The PLNR is located at the periphery of the renal lobules, while the ILNR is located in the central part of the lobe and tends to develop earlier.

Nephroblastomatosis is characterized by the presence of multifocal NRs and can present in different histological forms such as incipient, dormant, regressive, sclerotic, obsolescent and hyperplastic.

It is crucial to distinguish NR from WT, as clinical management differs significantly. NR typically does not have a fibrous pseudocapsule, which is almost always present in WT, but this distinction can be complicated in patients treated with preoperative chemotherapy. In some cases, the term “nephroblastic process, consistent with WT or NR” is used until further radiological-pathological data are available.

### Treatment

Treatment planning by a multidisciplinary team with experience in treating children with WT consisting of a pediatric surgeon and/or a pediatric urologist, a pediatric radiation oncologist, a pediatric oncologist and a pathologist is essential to define the appropriate treatment.

Most clinical studies on WT in children are conducted by COG RTC and SIOP, with different approaches (Table 2). Specifically, the COG RTC uses immediate surgery for unilateral tumors, while the SIOP begins with preoperative chemotherapy. Both include postoperative chemotherapy and, in advanced stages, risk-based radiotherapy, and infants younger than 6 months are treated with primary nephrectomy [24]. 

The COG RTC group, which includes the former NWTS group, has established the standard of treatment for WT in North America. This approach involves an initial nephrectomy, followed by chemotherapy and, in some cases, radiation. This method allows for a timely and precise histological diagnosis, the collection of biological materials not influenced by the therapy and the evaluation of staging, such as the presence of tumor leakage or lymph node involvement, before chemotherapy [25–27]. 

SIOP uses preoperative chemotherapy for patients with renal tumors before definitive resection. This method reduces tumor leakage during surgery and lowers the postoperative stage. Comparing histological features, preoperative chemotherapy alters tumor histology, reducing blastemal and mixed histology types, and results in fewer stage III tumors than immediate surgery [25–27]. 

**Table 2:** Wilms tumor treatment

Type	Characteristics
*Classic therapy* based on stage, according to Groups COG RTC and SIOP	*COG RTC*: immediate surgery for unilateral tumors; followed by chemotherapy, and in some cases, radiotherapy *SIOP*: preoperative chemotherapy to reduce the risk of tumor spillage and lower postoperative stage
*Stage I* Treatment varies based on tumor histology and patient-specific characteristics	*Standard*: nephrectomy with lymph node sampling, followed by chemotherapy with vincristine and dactinomycin for 18 weeks *With loss of heterozygosity on 1p/16q*: vincristine, dactinomycin, and doxorubicin for 24 weeks *Focal/Diffuse anaplastic tumor*: vincristine, dactinomycin, and doxorubicin for 24 weeks + radiotherapy
*Stage II* Several standardized options depending on tumor histology	*Favorable histology*: nephrectomy with lymph node sampling, followed by chemotherapy with vincristine and dactinomycin for 18 weeks *With loss of heterozygosity on 1p/16q*: vincristine, dactinomycin, and doxorubicin for 24 weeks *Focal anaplastic histology*: chemotherapy, abdominal radiotherapy *Diffuse anaplastic histology*: additional radiotherapy treatments
*Stage III* Combination of surgery, chemotherapy, and radiotherapy, varying by histological type and presence of specific genetic abnormalities	*Favorable histology*: nephrectomy with lymph node sampling, abdominal radiotherapy, and chemotherapy with vincristine, dactinomycin, doxorubicin for 24 weeks *With loss of heterozygosity on 1p/16q*: additional treatment with cyclophosphamide and etoposide *Focal/Diffuse anaplastic histology*: options for pre- and post-operative treatments, including abdominal radiotherapy
*Stage IV* Varies based on histological features and presence of specific genetic alterations	*Standard*: nephrectomy with lymph node sampling, abdominal and lung radiotherapy, chemotherapy with various regimens (e.g., vincristine, dactinomycin, doxorubicin, cyclophosphamide, etoposide) *Favorable histology with isolated lung nodules*: possible to avoid lung radiotherapy based on chemotherapy response *Anaplastic histology*: specific treatments based on response
*Stage V* No standard approach for the treatment of bilateral WT	*Standard*: preoperative chemotherapy, surgical resection, possible kidney transplant Nephron-sparing surgery to preserve renal function Postoperative chemotherapy customized based on tumor response and histology
*New therapies* Molecularly targeted agents, immunotherapies, genetic and epigenetic interventions	*Targeted therapies*: IGF1R inhibitors (e.g., BMS-754807, NVP-AEW541), VEGF/VEGFR inhibitors (e.g., apatinib, bevacizumab), PI3K/AKT pathway inhibitors (e.g., buparlisib, everolimus) *Immunotherapy*: COX-2 inhibitors, CAR-T cell therapy, MTAA-CTL *Genetic/Epigenetic therapies*: modifying miRNA profiles to influence tumor growth

#### Stage I

Treatment options for stage I WT (Table 2) patients vary based on tumor histology and patient-specific characteristics [24].

Children older than 24 months with tumor weighing more than 550 g treatment involves nephrectomy with lymph node sampling, followed by the chemotherapy regimen with vincristine and dactinomycin × 18 weeks after nephrectomy [28].

Patients with loss of heterozygosity on 1p/16q treatment involves nephrectomy with lymph node sampling, followed by the chemotherapy regimen with vincristine, dactinomycin and doxorubicin × 24 weeks [28].

Patients with focal anaplastic tumor treatment involves a nephrectomy with lymph node sampling, followed by a chemotherapy regimen with vincristine, dactinomycin and doxorubicin × 24 weeks and radiotherapy [28].

Patients with diffuse anaplastic cancer, treatment involves a nephrectomy with lymph node sampling, followed by a chemotherapy regimen with vincristine, dactinomycin and doxorubicin × 24 weeks and radiotherapy [28].

#### Stage II

Treatment of stage II WT (Table 2) involves several standardized options depending on the histology of the tumor [24].

For patients with favourable histology, the standard treatment consists of a nephrectomy accompanied by lymph node sampling, followed by the chemotherapy regimen with vincristine and dactinomycin × 18 weeks after nephrectomy [28].

In the case of patients with loss of heterozygosity on chromosomes 1p and 16q, the chemotherapy regimen with vincristine, dactinomycin and doxorubicin × 24 weeks [28].

For tumors with focal anaplastic histology, treatment involves nephrectomy and lymph node sampling, followed by abdominal radiotherapy and a chemotherapy regimen of vincristine, dactinomycin, and doxorubicin × 24 weeks [28].

Patients with diffuse anaplastic histology receive nephrectomy and lymph node sampling, followed by abdominal radiotherapy and chemotherapy regimen with vincristine, doxorubicin, cyclophosphamide, carboplatin, and etoposide × 30 weeks and with radiotherapy [29].

#### Stage III

Treatment of stage III WT (Table 2) involves a combination of surgery, chemotherapy and radiotherapy, with different options based on the histological type of the tumor and the presence or absence of particular genetic abnormalities, such as loss of heterozygosity of chromosomes 1p or 16q [24].

In all patients with favourable histology, the standard treatment involves nephrectomy with lymph node sampling, followed by abdominal radiotherapy and chemotherapy regimen with vincristine, dactinomycin, doxorubicin × 24 weeks [28].

Favourable histology with loss of heterozygosity of 1p and 16q, treatment consists of nephrectomy, lymph node sampling, abdominal radiation therapy and chemotherapy regimen with vincristine, dactinomycin, doxorubicin, cyclophosphamide and etoposide with subsequent radiation therapy [30].

In patients with focal anaplastic histology, it comprises two options. Post-operative treatment with lymph node sampling followed by abdominal radiotherapy and chemotherapy regimen with vincristine, dactinomycin and doxorubicin × 24 weeks [28]. Pre-operative treatment with chemotherapy regimen with vincristine, dactinomycin and doxorubicin × 24 weeks [28] followed by nephrectomy and abdominal radiotherapy.

Patients with diffuse anaplastic histology includes two options. Pre-operative treatment with vincristine, doxorubicin, cyclophosphamide, etoposide × 24 weeks [28] followed by nephrectomy, lymph node sampling and abdominal radiotherapy.

Immediate post-operative treatment with lymph node sampling, abdominal radiotherapy, and chemotherapy regimen with vincristine, doxorubicin, cyclophosphamide, carboplatin, and etoposide × 30 weeks + radiotherapy [30].

Timely initiation of radiotherapy within 14 days is fundamental for the multimodal treatment of patients with non-metastatic WT, delay is associated with an increased risk of mortality [24].

Negative lymph nodes and the absence of loss of heterozygosity are predictors of excellent survival rates, whereas patients with positive lymph nodes at the time of nephrectomy have a worse prognosis [24].

#### Stage IV

Treatment of stage IV WT (Table 2) varies depending on histological features and the presence of specific genetic alterations such as loss of heterozygosity on 1p/16q or gain of 1q [24].

Options include nephrectomy with lymph node sampling, abdominal and lung radiotherapy, and chemotherapy with regimens such as vincristine, dactinomycin, doxorubicin × 24 weeks [28] or vincristine, dactinomycin, doxorubicin, cyclophosphamide and etoposide with subsequent radiotherapy [30] in case of favourable histology with isolated lung nodules. If focal anaplasia regimen vincristine, dactinomycin, doxorubicin × 24 weeks [28]. If diffuse anaplasia regimen vincristine, doxorubicin, cyclophosphamide, etoposide × 24 weeks after nephrectomy [31].

Pulmonary radiotherapy may be avoided in some patients, depending on the initial response to chemotherapy. Liver metastases are not an independent adverse prognostic factor, and the use of liver surgery remains controversial.

#### Stage V

There is no standard approach for the treatment of bilateral WT stage V. One study has proposed guidelines, aiming to eliminate tumors and preserve kidney tissue to reduce the risk of chronic kidney disease [32].

Patients with bilateral cancer have worse survival rates than unilateral ones. Treatment options include preoperative chemotherapy, surgical resection, and, in some cases, kidney transplant. Nephron-sparing surgery is preferable to preserve renal function. Postoperative chemotherapy is customized based on tumor response and histology.

#### New Therapeutic Perspectives

New therapies for WT (Table 2) include molecularly targeted agents, immunotherapies, and genetic and epigenetic interventions [33, 34].

#### Molecular Targeted Therapy

The insulin-like growth factor 2 (IGF2) signaling pathway is closely linked to WT development [33]. Overexpression of IGF2, caused by loss of function of the DIS3L2 gene or mutations in some miRNAs that regulate PLAG1, contributes to the formation of this tumor. IGF1R, the IGF2 receptor, is considered a major therapeutic target due to its role in tumor growth. IGF1R inhibitors, such as BMS-754807 and NVP-AEW541, are being tested and show potential in reducing tumor growth in animal models [34].

WT requires angiogenesis to grow and spread, so the VEGF/VEGFR pathway is a common therapeutic target, with drugs such as apatinib, bevacizumab, and AZD2171 being used or tested to reduce the density of tumor blood vessels [34]. Control of WT1 could modulate the efficacy of therapy. However, anti-angiogenic therapy can cause serious side effects, such as pneumothorax, particularly in children [34].

The PI3K/AKT pathway is important for cell proliferation and survival, and abnormal activation may favor WT [33]. Inhibitors like buparlisib and everolimus can inhibit tumor growth, although buparlisib has high toxicity [34]. Modulating regulators such as PTEN and KRAS could offer new therapeutic strategies.

#### Immunotherapy

Immunotherapy aims to control and eliminate tumor cells by restoring or enhancing the normal immune response against the tumor.

Inhibition of COX-2 could be crucial in the treatment of WT. In mouse models, COX-2 is highly expressed in the tumor microenvironment, resulting in infiltration of immunosuppressive cells and production of cytokines such as IL-10 and TGF-β [35]. These factors promote tumor immune escape and thus COX-2 inhibition could be crucial in the treatment of WT.

CAR-T cell therapy uses CRISPR/Cas9 gene editing to modify T cells, allowing them to recognize and attack specific cancer cells. There are several generations of CAR-Ts, each with improvements in antigen recognition and immune response. However, the application of CAR-T for Wilms tumor is still in the experimental stage and the most promising target for it is B7-H3 [35, 36].

MTAA-CTL is an advanced cell therapy that uses CD8 + NK-T cells and CTLs to fight tumors more effectively than CAR-T therapy. This therapy uses dendritic cells modified with tumor-specific antigens to target multiple tumor antigens at the same time. Clinical trials have shown that it is safe and can improve response in WT patients, although further studies are needed to confirm its specific efficacy for this tumor [35, 37].

#### Genetic and Epigenetic Therapy

miRNA is a non-coding RNA that regulates gene expression post-transcriptionally by binding to mRNA and influencing translation or degradation of mRNA. Studies have shown significant differences in miRNA expression profiles between Wilms tumor cells and normal kidney tissues [34].

Several miRNAs such as miR-21, miR-19b, miR-483-3p, miR-891b, miR-613, miR-140-5p and miR-572 have been associated with the onset and development of WT [38, 39], and some of these miRNAs promote tumor growth, while others have suppressive effects [40].

## Non-Wilms Tumor

### Renal Clear Cell Sarcoma

Renal clear cell sarcoma (RCCS) is a rare malignant tumor that occurs in childhood characterized by aggressive behaviour with a tendency to metastasize and relapse [41].

RCCS is the second most frequent renal malignant neoplasm after WT [42] and represents 3% of pediatric tumors, with a peak incidence between 3 and 5 years of age and a male predominance of 2 to 1 [43] and approximately 4–12% present with metastatic disease at diagnosis [44].

RCCS is more aggressive than WT, is classified as a high-risk renal neoplasm with unfavourable histology and belongs to the group of “non-Wilms” renal tumors [45], tends to metastasize and late recurrences to the bones and brain, and is not associated with syndromes predisposing conditions [42].

Genetically, RCCS presents two main unique genetic aberrations such as internal tandem duplications in the BCOR gene [46] and the t(10;17) translocation involving the YWHAE and NUTM2B/E genes [47].

Frequently RCCS manifests as abdominal mass, abdominal pain, and hematuria. To diagnose RCCS, we usually start with an abdominal ultrasound to detect the abdominal mass. Next, a computed tomography scan and magnetic resonance imaging are performed to obtain detailed images and evaluate the extent of the tumor. A bone scan or PET/CT scan may be used to check for metastases.

Due to its significant similarity to WT, RCCS is often difficult to distinguish both clinically and radiographically and is often misdiagnosed in children due to morphological variability and absence of specific diagnostic markers [41].

However, it is essential to accurately differentiate RCCS and WT, as the treatments are different and correct diagnosis is crucial to improve prognosis and overall outcomes [41].

#### Pathology

##### Macroscopy

On macroscopic examination, RCCS is characterized by a large, well-circumscribed, unicentric mass, with a mean size of 11.3 cm and ranging from 2.3 to 24 cm. On section, it appears homogeneous light brown in color and has a sparkling mucinous appearance, frequently with dominant cystic foci and foci of necrosis and hemorrhage [47]. 

##### Histology

There are several morphological patterns that can coexist in variable proportions within the same tumor.

The classic pattern of RCCS is present in over 90% of cases [48] and is characterized by nests or cords of cells separated by fibrovascular septa, which can be delicate and branched or more robust and cellular, usually there are from 4 to 10 cells between vascular septa, with clear cytoplasm and round-oval nuclei with fine chromatin [47].

An important feature is the presence of finely dispersed hypochromic chromatin, and the cells may have “Orphan Annie” nuclei [48] and the intermediate matrix is composed of mucopolysaccharides. Furthermore, cell growth can trap renal tubules at the periphery of the lesion, displaying a regenerative appearance, similar to neoplastic tubules in WT [47].

The myxoid pattern characterized by variable pools of extracellular mucoid material containing hyaluronic acid highlighted by histochemical Alcian blue staining, which can lead to the formation of pseudocysts.

The sclerosing pattern characterized by intercellular deposition of dense, eosinophilic and collagenous material, which often gives an Indian file appearance to the umbilical cord cells which are organized into small nodules with reduced extracellular matrix, overlapping nuclei and frequent mitoses. This pattern is similar to the primary neuroectodermal tumor or blastemal component of WT.

The epithelioid pattern is characterized by umbilical cord cells with prominent eosinophilic cytoplasm and a trabecular or acinar growth pattern.

The palisade pattern or “verocay body” is characterized by fused cord cells, arranged in parallel linear arrays, perpendicular to the vascular septa, particularly umbilical cord or septal cells may take on a fused appearance and show random growth patterns or storiforms.

The anaplastic pattern is rare and is characterized by the presence of nuclear hyperchromatic, bizarre nuclei, and atypical mitoses.

##### Immunohistochemistry

RCCS is nonspecifically positive for vimentin, nerve growth factor receptor, cyclin D1, TLE1, SATB2, BCL-2, and CD10, and is negative for cytokeratins, epithelial membrane antigen, synaptophysin, S100, CD34, desmin, CD99 and WT1 [47].

Currently, strong and diffuse nuclear staining with BCL-6 coreceptor (BCOR) antibody is a specific marker useful for differentiating RCCS from other PCTs [49].

#### Treatment

Current international guidelines for RCCS treatment include the 2016 European UMBRELLA International Society of Pediatric Oncology (SIOP)-RTSG protocol and the North American NWTS-Children’s Oncology Group (COG) guidelines [50].

According to the SIOP protocol, preoperative chemotherapy is essential, whereas according to the NWTS-COG guidelines, primary surgery is essential.

Therapeutic indications vary based on the stage of the tumor. According to the current SIOP protocol, preoperative chemotherapy with actinomycin and vincristine is provided for tumors in stages I, II and III. For stage IV tumors, doxorubicin is also added. After initial surgical resection, postoperative chemotherapy treatment involves the use of etoposide, carboplatin, and ifosfamide, alternating with cyclophosphamide and doxorubicin. In addition, abdominal radiation therapy is provided for tumors in stages II, III and IV. Recurrences occur late so long follow-up is required.

### Renal Cell Carcinoma

Renal cell carcinoma (RCC) is very rare in children and accounts for approximately 2–6% of all pediatric renal tumors and with an incidence of 0.1-0.3% among all pediatric neoplasms [51], and and with peak incidence between 9 and 15 years [52].

Clinically, RCC most frequently manifests with an abdominal mass and gross hematuria, and less frequently with abdominal or flank pain, dysuria and urinary retention, and generalized symptoms such as fever, anemia, malaise, and weight loss, but can also be asymptomatic delaying diagnosis [51].

To diagnose RCCs, we usually start with an abdominal ultrasound to locate the mass in the abdomen. Next, a computed tomography and magnetic resonance imaging scan are performed to obtain detailed images and evaluate the extent of the tumor. A bone scan or PET/CT may be used to check for metastases.

RCC belongs to the group of pediatric kidney tumors known as “non-Wilms” and includes a wide range of subtypes. Among these, familial microphthalmia translocation renal cell carcinoma (MiT-RCC) is particularly common in children and adolescents [53, 54].

MiT-RCC, recognized by the WHO in 2004, is characterized by translocations involving the TFE3 or TFEB transcription factors, belonging to the MiT family [55]. It is the most common type of renal cell carcinoma in children, with the most common translocation affecting the TFE3 gene located on Xp11.2 [56].

Other histologic types of RCC described in children include papillary RCC (PRCC), renal medullary carcinoma (RMC), chromophobe RCC (CRCC), clear cell RCC (CCRCC),

Fumarate Hydratase-Deficient RCC, Succinate Dehydrogenase-Deficient RCC, Tuberous sclerosis-associated RCC, *ALK*-rearranged RCC, Thyroid-like RCC, Myoepithelial carcinoma and RCC not otherwise specified.

Fundamental in the diagnostic process are histology, immunohistochemistry and genetic analysis to confirm the subtypes of RCC and therefore choose the most suitable treatment.

#### Pathology

##### Macroscopy

On macroscopic examination, RCC is characterized by variable size, with a yellow to white color, both solid and cystic in consistency, and in more advanced cases it is common to find areas of necrosis and hemorrhages, the margins may be well delineated or infiltrative.

##### Histology, Immunohistochemistry and Genetics

MiT-RCC is characterized by a combination of nested and papillary architecture, variable amounts of clear and eosinophilic cytoplasm, and psammoma bodies, with two cytologic patterns, one with bulky cytoplasm and well-defined cell membranes, and one with smaller cells with less distinct cell membranes. Furthermore, T-RCC with a TFEB fusion transcript is characterized by a biphasic cell population consisting of large and small cells, with rearrangements of the TFEB gene highlighted by FISH. Instead, TFE3 MiT-RCC with strong widespread nuclear immunohistochemical expression of TFE3 and/or genetic confirmation of TFE3 gene rearrangement [56]. 

PRCC is classified into type 1 and type 2. PRCC type 1 is characterized by papillary structures lined by a single layer of cuboidal epithelium with little cytologic atypia and scant cytoplasm. PRCC type 2 is characterized by pseudostratification of the papillae, higher degrees of cytologic atypia, and more abundant eosinophilic cytoplasm [56, 57]. 

RMC is characterized by cells with eosinophilic cytoplasm and high nuclear grade arranged into tubular and reticular structures with an infiltrative growth pattern, associated with variable desmoplasia and neutrophilic inflammation [56].

CRCC is characterized by cells with pale eosinophilic cytoplasm arranged in a mostly solid pattern, with nuclei with wrinkled chromatin and perinuclear halos in variable degrees with immunohistochemistry positive for CD117 [56].

CCRCC is characterized by clear cells arranged in a solid growth pattern, possibly associated with known von Hippel-Lindau disease or multiple endocrine neoplasia syndrome type 1 [56, 57].

RCC with fumarate hydratase deficiency is characterized by cells displaying prominent nucleoli and clear perinucleolar halos lining papillary structures, with mutations in the fumarate hydratase gene [56].

RCC deficient in succinate dehydrogenase is characterized by cells with eosinophilic cytoplasm and intracytoplasmic vacuoles containing pale eosinophilic material, with germinal mutation in the succinate dehydrogenase B gene [56].

RCC associated with tuberous sclerosis is characterized by cells with granular eosinophilic cytoplasm in a solid, nested growth pattern [56].

ALK-rearranged RCC is characterized by cells containing abundant eosinophilic cytosol, intracytoplasmic lumina, and vesicular chromatin [56].

Thyroid RCC with follicular structures is characterized by numerous tubular/follicular structures containing dense eosinophilic material reminiscent of thyroid follicles [56].

Myoepithelial carcinoma is characterized by small epithelioid cells embedded in a myxoid stroma [56].

#### Treatment

Nephrectomy, whether radical or partial, is the mainstay treatment for pediatric RCC, with many patients requiring no adjuvant therapy after complete resection of localized disease [58].

Local lymph node involvement in children does not appear to adversely affect the outcome, while the need for radical lymph node dissection is still uncertain and should be considered in selected cases [58].

Survival in pediatric patients with RCC in stages I and II is greater than 90% but decreases significantly in stage III up to 60% and in stage IV up to 30% [59].

For unresectable metastatic RCC in children, treatment follows similar guidelines as for adults [60]. In particular, high-dose interleukin-2 appears to be effective for clear cell RCC, VEGF-targeted therapy with sunitinib and MET receptor tyrosine kinase inhibition in cases of RCC translocation, mTOR pathway inhibitors such as everolimus and temsirolimus in the stabilization of the disease [59]. Finally, chemotherapy is rarely indicated, but responses to regimens with gemcitabine, doxorubicin and oxaliplatin have been observed [59].

### Malignant Rhabdoid Tumour of the Kidney

Malignant rhabdoid tumor of the kidney (MRTK) is a rare tumor which can arise from embryonic stem cells and due to its high malignancy, aggressiveness and rapid progression, the prognosis for affected children is generally poor [61, 62].

MRTK is a highly malignant tumor that develops in the kidney, predominantly in newborns under 12 months of age and represents approximately 0.9-2% of pediatric renal tumors [61].

The initial clinical manifestations of MRTK can mimic that of WT [62] as abdominal mass and abdominal pain, and frequently metastasizes to the CNS [63].

The diagnostic process is the same as for other N-WTs.

#### Pathology

Histologically, MRTK is characterized by the presence of rhabdoid cells and usually by the deletion or mutation of the expression of the SMARCB1 gene also known as INI1 on immunohistochemistry [64].

In most cases, skeletal muscle markers such as desmin and myogenin are detectable, other markers such as CK, EMA and vimentin show positive expression, the expression of S-100, NSE and myoglobin proteins is variable, and the index of Ki67 proliferation is greater than 50% [64].

#### Treatment

The SIOP group’s treatment recommendations for MRTK include adjuvant chemotherapy with ifosfamide, etoposide, carboplatin, and doxorubicin, combined with radiotherapy [65, 66]. If the diagnosis of MRTK is made by biopsy before nephrectomy, intensive preoperative chemotherapy can be opted for instead of conventional preoperative chemotherapy with vincristine and dactinomycin [65].

The COG Panel recommends aggressive chemotherapy with cyclophosphamide, etoposide, and carboplatin, in addition to radiation therapy to the tumor bed, for all stages of the disease [65, 67].

### Congenital Mesoblastic Nephroma

Congenital mesoblastic nephroma (CMN) is a rare tumor with a mostly benign course and curable with early surgical intervention [68].

CMN is the most frequent renal tumor in the fetus and newborn, accounting for up to 87% of all renal tumors in the first 2 months of life, and the incidence decreases rapidly with increasing age, with WT becoming more common after 6 months of age [69].

In the prenatal phase, CMN clinically manifests itself with polyhydramnios, premature birth and neonatal hypertension, and is particularly difficult to diagnose both by ultrasound and magnetic resonance imaging, so it is often diagnosed in the third trimester [70].

In newborns, CMN most commonly presents as an intra-abdominal mass asymptomatic and may be associated with paraneoplastic syndromes such as hypertension and hypercalcemia [71], and ultrasound is useful for diagnosis.

On ultrasound, classic CMN appears as a solid homogeneous mass, characterized by a “ring” sign with concentric hypoechoic and hyperechoic borders surrounding the tumor [72]. In contrast, the cellular variant appears heterogeneously, showing fluid-containing spaces indicating hemorrhage, necrosis, and cystic changes [72].

#### Pathology

CMN is classified into three subtypes: cellular, classic and mixed. Among the histological variations of CMN, the cellular variant is the most frequent with 66%, followed by the classic variant 24% and the mixed subtype 10% [71].

On macroscopic examination, classic CMN is characterized by a solid gray-white tumor with a spiral appearance, while in cellular CMN the mass is soft and cystic with areas of hemorrhage and necrosis [71].

Classic CMN tumors resemble infantile myofibromatosis with fibroblastic cells, elongated nuclei, and low mitotic activity [73]. The cellular variant of CMN, on the other hand, is more cellular and resembles infantile fibrosarcoma, with ovoid cells, vesicular nuclei, high mitotic activity, and areas of necrosis and hemorrhage [73]. Mixed CMN combines features of both forms.

#### Treatment

The main treatment for CMN is radical nephrectomy, which is usually sufficient, in fact only approximately 5% of patients develop recurrences, due to the completeness of the resection [74].

The risk of recurrence is greater in patients over 3 months with the cellular variant of CMN and in cases with positive surgical margins or tumor rupture during surgery [75].

These cases, including relapses, may benefit from adjuvant chemotherapy, with combinations of vincristine, cyclophosphamide, and doxorubicin or vincristine, doxorubicin, and actinomycin D [75].

Cellular CMN is aggressive and can present malignancy, recurrence and metastasis, in contrast, patients with classic CMN generally have an excellent prognosis [76].

## Conclusions

In summary, pediatric renal tumors require a multidisciplinary approach to ensure precise diagnoses and effective treatments. Collaboration between specialists from different areas is crucial to personalize therapies and optimize clinical results, thus improving the quality of life of young patients and their families.

Advances in scientific research and the development of therapeutic technologies are continually evolving the recovery prospects for pediatric renal tumors. This advancement allows for the emergence of innovative approaches and increasingly precise therapeutic strategies, significantly improving treatment options and the chances of a favorable outcome for young patients.

### Key References

- Schulpen, M.; Roy, P.; Wijnen, M. H. W. A.; et al. Incidence and survival of paediatric renal tumours in the Netherlands between 1990 and 2014. European Journal of Cancer. 2022, 175, 282–290.
  - This study analyzes incidence and survival data of pediatric renal tumors in the Netherlands, providing useful information for international comparisons and historical evaluations of treatment strategies.
- Vujanić, G. M.; Mifsud, W.; Chowdhury, T.; et al. Characteristics and outcomes of preoperatively treated patients with anaplastic Wilms tumors registered in the UK SIOP-WT-2001 and IMPORT study cohorts (2002–2020). Cancer. 2022, 128(8), 1666–1675.
  - This study delves into the treatment and outcomes of patients with Wilms anaplastic tumor, highlighting significant advances in preoperative management and the prognostic impacts of new strategies.
- Libes, J.; Hol, J.; Neto, J. C. A.; et al. Pediatric renal tumor epidemiology: Global perspectives, progress, and challenges. Pediatric Blood & Cancer. 2023, 70 Suppl 2, e30343.
  - This review explores the global perspectives, progress and challenges in the management of pediatric renal tumors, contributing to an updated view of the state of the art.
- Avčin, S. L.; Črepinšek, K.; Jenko, Bizjan, B.; et al. Integrative Transcriptomic Profiling of the Wilms Tumor. Cancers. 2023, 15(15), 3846.
  - This study uses the integrated transcriptomic approach to gain insights into the molecular biology of Wilms tumor, opening new perspectives for the development of biomarkers and targeted therapies.
- Vujanić, G. M.; Mifsud, W. Anaplasia in Wilms tumor: A critical review. Pediatric Blood & Cancer. 2024, 71(7), e31000.
  - This article provides a critical review on anaplasia in Wilms tumor, delving into the prognostic impact and therapeutic implications of this rare but clinically relevant histological variant.
- Pasam, M. K.; Rao, B. V.; Chaganty, S. K.; et al. Grossing to reporting of Wilms tumor with emphasis on proper sampling in treatment-naive and postchemotherapy specimens and their clinicopathological correlation with outcome. Urology Annals. 2024, 16(1), 87–93.
  - This study highlights the importance of correct sampling and analysis of Wilms tumor specimens, both before and after chemotherapy, correlating pathological characteristics to clinical outcomes.

## Data Availability

No datasets were generated or analysed during the current study.

## References

[1] Libes J, Hol J, Neto JCA, Vallance KL, Tinteren HV, Benedetti DJ, Villar GLR, Duncan C, Ehrlich PF. Pediatric renal tumor epidemiology: global perspectives, progress, and challenges. Pediatric blood & cancer. 2023, 70 Suppl 2, e30343. 10.1002/pbc.30343 37096796

[2] Nakata K, Colombet M, Stiller CA, Pritchard-Jones K, Steliarova-Foucher E, Contributors I. Incidence of childhood renal tumours: an international population-based study. Int J Cancer. 2020;147(12):3313–27. 32902866 10.1002/ijc.33147 PMC7689773

[3] Qureshi SS, Bhagat M, Verma K, et al. Incidence, treatment, and outcomes of primary and recurrent non-wilms renal tumors in children: report of 109 patients treated at a single institution. J Pediatr Urol. 2020;16(4):e4751–9. 10.1016/j.jpurol.2020.05.168 32620510

[4] Jain J, Sutton KS, Hong AL. Progress Update in Pediatric Renal tumors. Curr Oncol Rep. 2021;23(3):33. 33591402 10.1007/s11912-021-01016-y

[5] Vujanić GM, Gessler M, Ooms AHAG, Collini P, Coulomb-l’Hermine A, D’Hooghe E, de Krijger RR, Perotti D, Pritchard-Jones K, Vokuhl C, van den Heuvel-Eibrink MM, Graf N, Study Group (SIOP–RTSG). The UMBRELLA SIOP-RTSG 2016 Wilms tumour pathology and molecular biology protocol. Nat Rev Urol. 2018;15(11):693–701. International Society of Paediatric Oncology–Renal Tumour. 30310143 10.1038/s41585-018-0100-3 PMC7136175

[6] Ward E, DeSantis C, Robbins A, Kohler B, Jemal A. Childhood and adolescent cancer statistics, 2014. CA Cancer J Clin. 2014;64(2):83–103. 24488779 10.3322/caac.21219

[7] Schulpen M, Roy P, Wijnen MHWA, Tytgat GAM, van den Heuvel-Eibrink MM, van Tinteren H, Karim-Kos HE. Incidence and survival of paediatric renal tumours in the Netherlands between 1990 and 2014. Eur J cancer (Oxford England: 1990). 2022;175:282–90. 10.1016/j.ejca.2022.08.021 36174300

[8] Leslie SW, Sajjad H, Murphy PB. Wilms Tumor. In StatPearls. StatPearls Publishing. 2023. 28723033

[9] Behjati S, Gilbertson RJ, Pfister SM. Maturation block in childhood cancer. Cancer Discov. 2021;11:542–4. 33589423 10.1158/2159-8290.CD-20-0926

[10] Spreafico F, Fernandez CV, Brok J, Nakata K, Vujanic G, Geller JI, Gessler M, Maschietto M, Behjati S, Polanco A, Paintsil V, Luna-Fineman S, Pritchard-Jones K. Wilms tumour. Nat Reviews Disease Primers. 2021;7(1):75. 34650095 10.1038/s41572-021-00308-8

[11] McMahon AP. Development of the mammalian kidney. Curr Top Dev Biol. 2016;117:31–64. 26969971 10.1016/bs.ctdb.2015.10.010 PMC5007134

[12] Huff V. Wilms’ tumours: about tumour suppressor genes, an oncogene and a chameleon gene. Nat Rev Cancer. 2011;11:111–21. 21248786 10.1038/nrc3002 PMC4332715

[13] Kitagawa K, Gonoi R, Tatsumi M, Kadowaki M, Katayama T, Hashii Y, Fujisawa M, Shirakawa T. Preclinical Development of a WT1 oral Cancer vaccine using a bacterial Vector to treat castration-resistant prostate Cancer. Mol Cancer Ther. 2019;18(5):980–90. 30824610 10.1158/1535-7163.MCT-18-1105

[14] Gadd S, Huff V, Walz AL, Ooms AHAG, Armstrong AE, Gerhard DS, Smith MA, Auvil JMG, Meerzaman D, Chen QR, Hsu CH, Yan C, Nguyen C, Hu Y, Hermida LC, Davidsen T, Gesuwan P, Ma Y, Zong Z, Mungall AJ, Perlman EJ. A children’s Oncology Group and TARGET initiative exploring the genetic landscape of Wilms tumor. Nat Genet. 2017;49(10):1487–94. 28825729 10.1038/ng.3940 PMC5712232

[15] Nakata K, Colombet M, Stiller CA, Pritchard-Jones K, Steliarova-Foucher E. Incidence of childhood renal tumours: an international population-based study. Int J Cancer. 2020;147:3313–27. 32902866 10.1002/ijc.33147 PMC7689773

[16] Cunningham ME, et al. Global disparities in Wilms tumor. J Surg Res. 2020;247:34–51. 31810638 10.1016/j.jss.2019.10.044

[17] Parkin DM, et al. Stage at diagnosis and survival by stage for the leading childhood cancers in three populations of sub-saharan Africa. Int J Cancer. 2021;148:2685–91. 33433927 10.1002/ijc.33468

[18] Saltzman AF, Cost NG, Romao RL. P. Wilms Tumor. Urologic Clin North Am. 2023;50(3):455–64. 10.1016/j.ucl.2023.04.008 37385707

[19] Popov SD, Sebire NJ, Vujanic GM. Wilms’ Tumour– Histology and Differential diagnosis. In: van den Heuvel-Eibrink MM, editor. Wilms Tumor. Codon; 2016. 27512769

[20] Breslow NE, Beckwith JB, Perlman EJ, Reeve AE. Age distributions, birth weights, nephrogenic rests, and heterogeneity in the pathogenesis of Wilms tumor. Pediatr Blood Cancer. 2006;47(3):260–7. 16700047 10.1002/pbc.20891 PMC1543666

[21] Vujanić GM, Sandstedt B. The pathology of Wilms’ tumour (nephroblastoma): the International Society of Paediatric Oncology approach. J Clin Pathol. 2010;63(2):102–9. 19687012 10.1136/jcp.2009.064600

[22] Vujanić GM, Mifsud W. Anaplasia in Wilms tumor: a critical review. Pediatr Blood Cancer. 2024;71(7):e31000. 38605554 10.1002/pbc.31000

[23] Vujanić GM, Mifsud W, Chowdhury T, Al-Saadi R, Pritchard-Jones K. Renal Tumour Special Interest Group of the children’s Cancer and Leukaemia Group. Characteristics and outcomes of preoperatively treated patients with anaplastic Wilms tumors registered in the UK SIOP-WT-2001 and IMPORT study cohorts (2002–2020). Cancer. 2022;128(8):1666–75. 35119702 10.1002/cncr.34107

[24] PDQ Pediatric Treatment Editorial Board. Wilms Tumor and Other Childhood Kidney Tumors Treatment (PDQ^®^): Health Professional Version. PDQ Cancer Information Summaries. National Cancer Institute (US); 2024. 26389282

[25] Nelson MV, van den Heuvel-Eibrink MM, Graf N, Dome JS. New approaches to risk stratification for Wilms tumor. Curr Opin Pediatr. 2021;33(1):40–8. 33394739 10.1097/MOP.0000000000000988 PMC7919941

[26] Pasam MK, Rao BV, Chaganty SK, Sharma RM, Patil V, Kodandapani S, Challa S, Thammineedi SR. Grossing to reporting of Wilms tumor with emphasis on proper sampling in treatment-naive and postchemotherapy specimens and their clinicopathological correlation with outcome. Urol Annals. 2024;16(1):87–93. 10.4103/ua.ua_60_23 PMC10896324 38415234

[27] Mergen M, Welter N, Furtwängler R, Melchior P, Vokuhl C, Gessler M, Meier CM, Kager L, Schenk JP, Graf N. The impact of the route to diagnosis in nephroblastoma. Cancer medicine. 2024; 13(10), e7226. 10.1002/cam4.7226 PMC11117195 38785181

[28] Grundy PE, Breslow NE, Li S, Perlman E, Beckwith JB, Ritchey ML, Shamberger RC, Haase GM, D’Angio GJ, Donaldson M, Coppes MJ, Malogolowkin M, Shearer P, Thomas PR, Macklis R, Tomlinson G, Huff V, Green DM. & National Wilms Tumor Study Group. Loss of heterozygosity for chromosomes 1p and 16q is an adverse prognostic factor in favorable-histology Wilms tumor: a report from the National Wilms Tumor Study Group. J Clin Oncology: Official J Am Soc Clin Oncol. 2005;23(29):7312–21. 10.1200/JCO.2005.01.2799 16129848

[29] Daw NC, Chi YY, Kalapurakal JA, et al. Activity of vincristine and irinotecan in diffuse Anaplastic Wilms Tumor and Therapy outcomes of Stage II to IV Disease: results of the children’s Oncology Group AREN0321 Study. J Clin Oncol. 2020;38(14):1558–68. 32134700 10.1200/JCO.19.01265 PMC7213587

[30] Dix DB, Seibel NL, Chi YY, et al. Treatment of Stage IV Favorable Histology Wilms Tumor with Lung metastases: a Report from the children’s Oncology Group AREN0533 Study. J Clin Oncol. 2018;36(16):1564–70. 29659330 10.1200/JCO.2017.77.1931 PMC6075846

[31] Dome JS, Cotton CA, Perlman EJ, et al. Treatment of anaplastic histology Wilms’ tumor: results from the fifth National Wilms’ Tumor Study. J Clin Oncol. 2006;24(15):2352–8. 16710034 10.1200/JCO.2005.04.7852

[32] Ehrlich P, Chi YY, Chintagumpala MM, et al. Results of the first prospective multi-institutional treatment study in children with bilateral Wilms Tumor (AREN0534): a Report from the children’s Oncology Group. Ann Surg. 2017;266(3):470–8. 28795993 10.1097/SLA.0000000000002356 PMC5629006

[33] Karam S, Gebreil A, Alksas A, Balaha HM, Khalil A, Ghazal M, Contractor S, El-Baz A. Insights into Personalized Care strategies for Wilms Tumor: a narrative literature review. Biomedicines. 2024;12(7):1455. 39062028 10.3390/biomedicines12071455 PMC11274555

[34] Hong B, Dong R. Research advances in the targeted therapy and immunotherapy of Wilms tumor: a narrative review. Translational cancer Res. 2021;10(3):1559–67. 10.21037/tcr-20-3302 PMC8799117 35116480

[35] Hont AB, Dumont B, Sutton KS, Anderson J, Kentsis A, Drost J, Hong AL, Verschuur A. The tumor microenvironment and immune targeting therapy in pediatric renal tumors. Pediatr Blood Cancer. 2023;70(Suppl 2):e30110. 36451260 10.1002/pbc.30110

[36] Majzner RG, Theruvath JL, Nellan A, et al. CAR T cells target-ing B7-H3, a pan-cancer antigen, demonstrate potent preclini-cal activity against pediatric solid tumors and brain tumors. ClinCancer Res. 2019;25(8):2560–74. 10.1158/1078-0432.CCR-18-0432 PMC8456711 30655315

[37] Hont AB, Cruz CR, Ulrey R, O’Brien B, Stanojevic M, Datar A, Albihani S, Saunders D, Hanajiri R, Panchapakesan K, Darko S, Banerjee P, Fortiz MF, Hoq F, Lang H, Wang Y, Hanley PJ, Dome JS, Bollard CM, Meany HJ. Immunotherapy of Relapsed and Refractory Solid Tumors with Ex vivo expanded Multi-tumor Associated Antigen Specific cytotoxic T lymphocytes: a phase I study. J Clin Oncology: Official J Am Soc Clin Oncol. 2019;37(26):2349–59. 10.1200/JCO.19.00177 PMC6804838 31356143

[38] Alfaifi J. miRNAs role in Wilms tumor pathogenesis: signaling pathways interplay. Pathol Res Pract. 2024;256:155254. 38460245 10.1016/j.prp.2024.155254

[39] Avčin SL, Črepinšek K, Jenko, Bizjan B, Šket R, Kovač J, Vrhovšek B, Blazina J, Blatnik O, Kordič R, Kitanovski L, Jazbec J, Debeljak M, Tesovnik T. Integrative transcriptomic profiling of the Wilms Tumor. Cancers. 2023;15(15):3846. 37568662 10.3390/cancers15153846 PMC10416970

[40] Zheng H, Liu J, Pan X, Cui X. Biomarkers for patients with Wilms tumor: a review. Front Oncol. 2023;13:1137346. 37554168 10.3389/fonc.2023.1137346 PMC10405734

[41] Shrestha AL, Shrestha P, Mishra A, Pandit A. A cystic non-wilms renal tumor in a Nepalese boy: a rare case of clear cell sarcoma. Int J Surg case Rep. 2023;109:108582. 37517259 10.1016/j.ijscr.2023.108582 PMC10400875

[42] Fiore M, Taddia A, Indio V, Bertuccio SN, Messelodi D, Serravalle S, Bandini J, Spreafico F, Perotti D, Collini P, Di Cataldo A, Pasquinelli G, Chiarini F, Fois M, Melchionda F, Pession A, Astolfi A. Molecular signature of Biological aggressiveness in Clear Cell Sarcoma of the kidney (CCSK). Int J Mol Sci. 2023;24(4):3743. 36835166 10.3390/ijms24043743 PMC9964999

[43] Aviral G, Sarvesh CM, Sushila J, Ansari MS. Clear cell sarcoma of kidney: a mimicker of Wilms’ tumor. J Cancer Res Ther. 2023;19(5):1468–70. 37787333 10.4103/jcrt.jcrt_432_21

[44] Zhang A, Yuan X, Jiang S, Xu D, Huang C, Tang JY, Gao Y. Outcomes of children with clear cell sarcoma of kidney following NWTS strategies in Shanghai China (2003–2021). PLoS ONE. 2024;19(7):e0306863. 38980838 10.1371/journal.pone.0306863 PMC11233012

[45] Laasri K, Bahlouli N, Chait F, Isfaoun Z, Hessissen L, Rouas L, Lamalmi N, Allali N, El Haddad S. Chat, L. A rare case of renal tumor in children: clear cell sarcoma with an unusual presentation. Radiol case Rep. 2023;18(11):3865–71. 37670910 10.1016/j.radcr.2023.08.013 PMC10475403

[46] Zhang M, Yao X, Guan X, Jia C, Zhang R, Wang H, Guo Y, Ni X, Yu Y, He L. Clinical relevance of BCOR internal tandem duplication and TP53 aberration in clear cell sarcoma of the kidney. Hum Pathol. 2023;134:45–55. 36563883 10.1016/j.humpath.2022.12.007

[47] Aldera AP, Pillay K. Clear cell sarcoma of the kidney. Arch Pathol Lab Med. 2020;144(1):119–23. 30917048 10.5858/arpa.2018-0353-RS

[48] Argani P, Perlman EJ, Breslow NE, et al. Clear cell sarcoma of the kidney: a review of 351 cases from the National Wilms Tumor Study Group Pathology Center. Am J Surg Pathol. 2000;24(1):4. 10632483 10.1097/00000478-200001000-00002

[49] Argani P, Pawel B, Szabo S, Reyes-Múgica M, Timmons C, Antonescu CR. Diffuse strong BCOR immunoreactivity is a sensitive and specific marker for clear cell sarcoma of the kidney (CCSK) in pediatric renal neoplasia. Am J Surg Pathol. 2018;42(8):1128–31. 29851702 10.1097/PAS.0000000000001089 PMC6041176

[50] Gooskens SL, Graf N, Furtwängler R, Spreafico F, Bergeron C, Ramírez-Villar GL, Godzinski J, Rübe C, Janssens GO, Vujanic GM, Leuschner I, Coulomb-L’Hermine A, Smets AM, de Camargo B, Stoneham S, van Tinteren H, Pritchard-Jones K, van den Heuvel-Eibrink MM. International Society of Paediatric Oncology–Renal Tumour Study Group (SIOP–RTSG). Position paper: Rationale for the treatment of children with CCSK in the UMBRELLA SIOP-RTSG 2016 protocol. Nat Rev Urol. 2028;15(5):309–19. 10.1038/nrurol.2018.14 29485128

[51] He M, Cai J, Zhu K, Gu W, Li M, Xiong J, Guan Z, Wang J, Shu Q. Renal cell carcinoma in children and adolescents: single-center experience and literature review. Medicine. 2021;100(2):e23717. 33466124 10.1097/MD.0000000000023717 PMC7808530

[52] Chaabouni A, Samet A, Fourati M, Mejdoub B, Kammoun O, Mseddi MA, Hadjslimene M. Renal cell carcinoma in children, report of a new case. Urol case Rep. 2021;39:101813. 34504772 10.1016/j.eucr.2021.101813 PMC8414171

[53] Khondker A, Kwong JCC, Chua ME, Kim JK, Chan JYH, Zappitelli M, Brzezinski J, Cost NG, Rickard M, Lorenzo AJ. Nephron-sparing surgery for renal cell carcinoma in children and young adults: a systematic review. Urol Oncol. 2023;41(3):137–44. 36428167 10.1016/j.urolonc.2022.09.015

[54] Denize T, Massa S, Valent A, Militti L, Bertolotti A, Barisella M, Rioux-Leclercq N, Malouf GG, Spreafico F, Verschuur A, van der Beek J, Tytgat L, van den Heuvel-Eibrink MM, Vujanic G, Collini P, Coulomb A. Renal cell carcinoma in children and adolescents: a retrospective study of a french-italian series of 93 cases. Histopathology. 2022;80(6):928–45. 35238063 10.1111/his.14634

[55] van der Beek JN, Hol JA, Coulomb-l’Hermine A, Graf N, van Tinteren H, Pritchard-Jones K, Houwing ME, de Krijger RR, Vujanic GM, Dzhuma K, Schenk JP, Littooij AS, Ramírez-Villar GL, Murphy D, Ray S, Al-Saadi R, Gessler M, Godzinski J, Ruebe C, Collini P, van den Heuvel-Eibrink MM. Characteristics and outcome of pediatric renal cell carcinoma patients registered in the International Society of Pediatric Oncology (SIOP) 93– 01, 2001 and UK-IMPORT database: a report of the SIOP-Renal Tumor Study Group. Int J Cancer. 2021;148(11):2724–35. 33460450 10.1002/ijc.33476 PMC8048605

[56] Cajaiba MM, Dyer LM, Geller JI, Jennings LJ, George D, Kirschmann D, Rohan SM, Cost NG, Khanna G, Mullen EA, Dome JS, Fernandez CV, Perlman EJ. The classification of pediatric and young adult renal cell carcinomas registered on the children’s oncology group (COG) protocol AREN03B2 after focused genetic testing. Cancer. 2018;124(16):3381–9. 29905933 10.1002/cncr.31578 PMC6108909

[57] Rao Q, Chen JY, Wang JD, Ma HH, Zhou HB, Lu ZF, Zhou XJ. Renal cell carcinoma in children and young adults: clinicopathological, immunohistochemical, and VHL gene analysis of 46 cases with follow-up. Int J Surg Pathol. 2011;19(2):170–9. 20034980 10.1177/1066896909354337

[58] Ray S, Jones R, Pritchard-Jones K, Dzhuma K, van den Heuvel-Eibrink M, Tytgat G, van der Beek J, Oades G, Murphy D. Pediatric and young adult renal cell carcinoma. Pediatr Blood Cancer. 2020;67(11):e28675. 32869954 10.1002/pbc.28675

[59] Brok J, Treger TD, Gooskens SL, van den Heuvel-Eibrink MM, Pritchard-Jones K. Biology and treatment of renal tumours in childhood. European journal of cancer (Oxford, England: 1990). 2026; 68, 179–195. 10.1016/j.ejca.2016.09.005 27969569

[60] Ljungberg B, Bensalah K, Canfield S, Dabestani S, Hofmann F, Hora M, Kuczyk MA, Lam T, Marconi L, Merseburger AS, Mulders P, Powles T, Staehler M, Volpe A, Bex A. EAU guidelines on renal cell carcinoma: 2014 update. European urology. 2015; 67(5): 913–924. 10.1016/j.eururo.2015.01.005 25616710

[61] Zhanghuang C, Zhang Z, Zeng L, Yan B, Tang H, Wang J, Liu X, Wei G, He D. Clinical and prognostic analysis of 42 children with malignant rhabdoid tumor of the kidney: a 7-year retrospective multi-center study. BMC Pediatr. 2022;22(1):591. 36229776 10.1186/s12887-022-03643-1 PMC9563785

[62] Xie S, Fang Y, Yang Y, Liu L, Bai J, Lin S, Zhang B, Fang Y. Extracranial malignant rhabdoid tumors in children: high mortality even with the help of an aggressive clinical approach. Eur J Pediatrics. 2024;183(2):557–67. 10.1007/s00431-023-05345-x 38019286

[63] Schenk JP, Hötker A, Furtwängler R, Fuchs J, Warmann SW, Graf N. Bildgebung renaler Tumoren Im Kindesalter [Imaging of renal tumors in children]. Radiologe. 2021;61(7):619–28. 34143242 10.1007/s00117-021-00864-w

[64] Li J, Zhang W, Hu H, Zhang Y, Wang Y, Gu H. Huang, D. Case Analysis of 14 children with malignant rhabdoid tumor of the kidney. Cancer Manage Res. 2021;13:4865–72. 10.2147/CMAR.S309274 PMC8232862 34188539

[65] Qureshi SS, Bhagat M, Verma K, Yadav S, Prasad M, Vora T, Chinnaswamy G, Amin N, Smriti V, Baheti A, Laskar S, Khanna N, Ramadwar M, Shah S. Incidence, treatment, and outcomes of primary and recurrent Non-Wilms renal tumors in children: Report of 109 patients treated at a single institution. Journal of pediatric urology. 2020; 16(4):, 475.e1–475.e9. 10.1016/j.jpurol.2020.05.168 32620510

[66] van den Heuvel-Eibrink MM, van Tinteren H, Rehorst H, Coulombe A, Patte C, de Camargo B, et al. Malignant rhabdoid tumours of the kidney (MRTKs), registered on recent SIOP protocols from 1993 to 2005: a report of the SIOP renal tumour study group. Pediatr Blood Canc. 2011;56:733–7. 10.1002/pbc.22922 21370404

[67] Tomlinson GE, Breslow NE, Dome J, Guthrie KA, Norkool P, Li S et al. Rhabdoid tumour of the kidney in the National Wilms’ Tumor Study: age at diagnosis as a prognostic factor. J Clin Oncol. 2005; 23: pp. 7641–7645. 10.1200/JCO.2004.00.8110 16234525

[68] Grosinger L, Salik I, Mehta B. Infantile congenital Mesoblastic Nephroma leading to multi-systemic end-organ disease. Cureus. 2022;14(10):e30513. 36415355 10.7759/cureus.30513 PMC9675395

[69] Rayner J, Vinycomb T, Wanaguru D, Jiwane A. Congenital mesoblastic nephroma: review of current management and outcomes in a single centre. ANZ J Surg. 2023;93(4):1008–11. 36382605 10.1111/ans.18165

[70] Che M, Yang F, Huang H, Zhang H, Han C, Sun N. Prenatal diagnosis of fetal congenital mesoblastic nephroma by ultrasonography combined with MR imaging: a case report and literature review. Medicine. 2021, 100(3), e24034. 10.1097/MD.0000000000024034 PMC7837828 33546001

[71] Simkhada A, Paudel R, Sharma N. Congenital Mesoblastic Nephroma: a Case Report. JNMA. 2023;61(259):259–62. 10.31729/jnma.7979 PMC10231538 37203959

[72] Chung EM, Graeber AR, Conran RM. Renal tumors of Childhood: radiologic-pathologic correlation part 1. The 1st Decade: from the Radiologic Pathology archives. Radiographics: Rev Publication Radiological Soc North Am Inc. 2016;36(2):499–522. 10.1148/rg.2016150230 26963460

[73] Ooms AHAG, Vujanić GM, D’Hooghe E, Collini P, L’Herminé-Coulomb A, Vokuhl C, Graf N, Heuvel-Eibrink MMVD, de Krijger RR. Renal tumors of Childhood-A histopathologic pattern-based Diagnostic Approach. Cancers. 2020;12(3):729. 32204536 10.3390/cancers12030729 PMC7140051

[74] Santos LG, Carvalho JdeS, Reis MA, Sales RL. Cellular congenital mesoblastic nephroma: case report. Jornal brasileiro de nefrologia. 2011;33(1):109–12. 21541470

[75] Ahmed HU, Arya M, Levitt G, Duffy PG, Mushtaq I, Sebire NJ, Part I. Primary malignant non-wilms’ renal tumours in children. Lancet Oncol. 2007;8(8):730–7. 17679083 10.1016/S1470-2045(07)70241-3

[76] De Wilde K, Zuberi J. Case report: congenital mesoblastic nephroma. Int J Surg case Rep. 2023;106:108233. 37141775 10.1016/j.ijscr.2023.108233 PMC10201815

